# Effective Ways to Encourage Health-Care Practices among Cultural Minorities in Israel during the COVID-19 Pandemic

**DOI:** 10.3390/ijerph18189563

**Published:** 2021-09-10

**Authors:** Tehila Kalagy, Sarah Abu-Kaf, Orna Braun-Lewensohn

**Affiliations:** 1 Department of Management and Public Policy, Ben-Gurion University of the Negev, Beer-Sheva 84105, Israel; 2 Conflict Management & Resolution Program, Department of Interdisciplinary Studies, Ben-Gurion University of the Negev, Beer-Sheva 84105, Israel; aks@bgu.ac.il (S.A.-K.); ornabl@bgu.ac.il (O.B.-L.)

**Keywords:** COVID-19, health policy, cultural minorities, community sense of coherence, Ultra-Orthodox Jews, Arab society

## Abstract

Following the worldwide outbreak of COVID-19, policymakers have been occupied with the questions of whether and how to specially address unique cultural groups coping with the pandemic. This study aimed to evaluate the potential for a culturally tailored approach to the transmission of health messages in a time of crisis among two minority populations within Israeli society: the Ultra-Orthodox population and the Arab population. To that end, 380 individuals from Israeli Ultra-Orthodox society and 360 individuals from Israeli Arab society completed a self-reported questionnaire in early April 2020, in the midst of the COVID-19 pandemic. The findings of this study reveal differences between these groups in terms of the effectiveness of different channels for conveying messages and the channels that were preferred, as well as significant relationships between community sense of coherence and the study variables. We found that advocacy and motivation based on values, on the one hand, and recognition of the effectiveness of a culturally tailored approach, on the other, may be the best approach for persuading members of minority populations, who belong to collectivist societies, to comply with epidemic-control instructions.

## 1. Introduction

COVID-19 has presented health-care systems with an emergency situation that involves an immediate need to provide appropriate responses, to slow the spread of the virus and to cope with the consequences of infection. During this crisis, policymakers have faced a need to convey messages to different ethnic-minority populations with unique cultural characteristics. In the current crisis, we have seen that the use of standard tools, such as messages delivered through media aimed at the general population without any distinction between different, culturally unique populations, is not sufficient to inspire the different populations in the country to answer the call to protect themselves from the disease [1]. Our goal in this study was to evaluate the inherent potential in a culturally tailored approach to messaging related to personal and public health during a severe health crisis, with a particular focus on the Ultra-Orthodox and Arab minorities in Israel.

Due to the fact that among both populations, the rates of infection have been high, it is very important of figuring out the best ways to reach these populations. The two populations are both characterized by collective, family and social values that encourage social connections and social contact, including group religious activities that may fuel the spread of the virus.

A study that examined housing density and the COVID-19 morbidity rate found a direct relationship between those two factors among these two groups. The density of housing among the Ultra-Orthodox population in Israel is 1.4 people per room, similar to the density among Arabs, and notably different from the overall figure for the Jewish Israeli population, which stands at 0.8. The combination of a high housing density and a large number of social interactions increases the risk of infection [2].

The decision to focus on these two populations was based on their position as the two largest minorities in the Israeli society and their unique social and cultural characteristics. The Ultra-Orthodox population in Israel is a religious, conservative minority population that has the fastest growth rate in developed countries [3,4]. According to Israel’s Central Bureau of Statistics, the Ultra-Orthodox population in Israel numbered one million in 2017, accounting for 12% of the total population at that time [5]. The Ultra-Orthodox community is expected to account for 16% of the population in 2030 [6]. The relatively low socio-economic level of this population is evident in its low rate of consumption of health services. The Ultra-Orthodox community demands that its members abide by the norms and the comprehensive social ethos dictated by the “Torah Giants of our Generation” [7], including attitudes toward government regulations, in general, and health-system guidelines, in particular.

Arabs are the largest minority group in Israel. At the end of 2019, the Arab population comprised about 21% of the total population. Compared to Israeli Jews, Arabs are disadvantaged in terms of almost every socioeconomic indicator. They have lower incomes, higher levels of unemployment and lower levels of educational attainment [8]. They face high rates of poverty and there are social and cultural gaps between them and the majority group [3]. The reasons for this group’s separation from the Jewish population are many and the results of this separation are manifested in huge disparities between it and the rest of Israeli society [9]. At the same time, traditional Arab society in Israel is undergoing a process of modernization and globalization [10]. Arab society is characterized by a large degree of power distance [11]. Inequalities based on gender and age are common; male over female and the older over the younger [12,13]. In recent years, as educational levels have increased among Arabs, in general, and the number of Arabs in the health professions has increased, in particular, an additional variable related to educational level has been added to these inequalities. Specifically, educated individuals now have more power over less-educated individuals [14].

Minority groups, especially those in conflict with the state, exhibit low levels of trust in state institutions [15]. Ultra-Orthodox society usually views state institutions with suspicion and has a long history of distrust of the authorities based on historical narratives that are at odds with the secular nature of the state of Israel. Among Arab society, there is a growing distrust of the state, mainly due to the national rift. The continuing Palestinian–Israeli conflict has left the Arab minority under considerable stress and subject to expressions of discrimination [16].

That said, over the years, Ultra-Orthodox society has undergone a process of accelerated integration, at both the political and social levels, a trend that has increased confidence in the state. In Arab society, the process has been different. There has been integration in employment and Higher education. But, politically, the parties who represent this public traditionally have less taken part in the governing coalition [15].

In light of the unique characteristics of these two minority populations, we wanted to identify the most common ways of listening to health messages among each of these populations during the COVID-19 crisis. We also wanted to identify the effects of gender and cultural-minority status on the efficacy of different ways of messaging during the pandemic. Finally, we wanted to explore the relationships between the different ways of messaging and community sense of coherence within each of the groups and determine whether those relationships are similar in the two groups.

### 1.1. Cultural Competency and the Conveyance of Messages during an Epidemic

Throughout history, humanity has had to cope with crises, including epidemics, and policymakers have evaluated strategies for effectively halting epidemics in broad cultural contexts [17]. In recent decades, the value of cultural diversity has been increasingly integrated into different areas of life, as expressed in the term cultural competency. According to this approach, it is important to be acquainted with the importance of the values and behavioral norms, the socio-political conditions, and the living conditions of different groups and to develop policies accordingly. This is particularly important in the field of health, in general, and in coping with an epidemic, in particular [18,19].

During a health crisis, there are risk factors that can amplify the injury to minority groups, such as living conditions, in general, and density levels, in particular, as well as a lack of culturally appropriate welfare and health services, limited access to health-care services, relatively low socioeconomic status, language and cultural barriers that affect how information and messages are received from authorities, and limited trust in the authorities and their response [1]. Another factor that can amplify this injury is the selective relationship between traditional groups and different media networks [20]. According to the cultural-competency approach, during public-health crises, the messages conveyed to citizens from different cultures, particularly different religious cultures, should be different from those conveyed to the general population [21].

Studies that have examined the role of religious authorities as compared to that of medical authorities have found that cooperative dialogue between medical authorities and religious groups is productive and eases the tension between religion and science [22,23]. The conveyance of culturally adapted messages is a partnership between those who set health policy and key figures in traditional communities who grant their approval for those policies [7,24].

Cultural competency in policy development also addresses issues of gender and the importance of gender differences in how individuals relate to health information [25]. It has been found that, due to cultural structures, men are less aware of health information and less motivated to locate such information. Women dedicate a lot more attention to staying up-to-date with medical information, especially during global epidemics [26]. Ek [27] argued that optimal public-health interventions should involve methods that are sensitive to the gender gap.

It is only in the last decade that the state of Israel, which includes minority populations characterized by cultural and language differences, has officially joined the Western world’s trend toward cultural competency [28]. At this time of the COVID-19 pandemic, the need to develop culturally appropriate policies is particularly acute.

### 1.2. Community Sense of Coherence in Emergencies

Sense of coherence (SOC) is a term coined by Antonovsky [29,30] that is based on the salutogenic model for health resources, with the intent of examining the question of how individuals cope with the harsh world around them [31]. The term community sense of coherence refers to the attitudes and perceptions of community members in relation to the community in which they live, with reference to the way those attitudes and perceptions capture the three components inherent to SOC: comprehensibility, manageability and meaningfulness [32]. Comprehensibility refers to those resources that foster an individual’s feeling that the community is a safe place for him or her, enabling the individual to predict both what will happen when he or she acts in a certain way and the community’s present and future directions. Community manageability refers to those resources that can help individuals in times of need and stress, such as good and accessible social services for children and youth. SOC has been found to be related to wellness in a variety of contexts [33]. Therefore, it fits nicely with the understanding of optimal health and the ability of an individual and his or her community to cope with crisis situations [34].

In view of the unique nature of the two cultural groups that were the focus of this study, we sought to examine the methods for conveying messages through a few key questions:

Who are the individuals to whom individuals are most likely to listen, with regard to messages about the pandemic?

What information makes it possible for individuals to follow epidemic-control instructions?

How should these messages be delivered?

When should these messages to be delivered?

We also wanted to identify the effects of gender and cultural group on the efficacy of different ways of messaging during the pandemic. Finally, we wanted to explore the relationships between the different ways of messaging and community SOC within each of the groups and determine whether those relationships are similar across the two groups.

## 2. Methods

### 2.1. Participants

In this case, 678 individuals participated in the current study. Age ranged between 18–67 years (*M* = 34.16; *SD* = 11.46). Women accounted for 58.3% of the sample. The participants were married (67.7%), single (28.3%), divorced (3.4%) or widowed (0.1%). As for income—66.4% reported below average income, 19.3% average income and 14.2% above average income.

### 2.2. Procedure

All of the ethical procedures applicable to this study were followed. The study was approved by the Human Subjects Ethics Committee of the Conflict Management and Resolution Program at Ben-Gurion University of the Negev (Approved Ethics Form No. 2020-004).

The participants were recruited by the Midgam panel (https://www.midgampanel.com/ (accessed on 1 December 2020)) and represent the various ethnic groups examined in this study. The Midgam panel gives each participant a small amount for appreciation of participation.

Participants completed self-report questionnaires in the beginning of April 2020, in the midst of the COVID-19 pandemic. The participants were informed that the researchers were interested in their experience and were also informed that participation was voluntary and anonymous, and that they were free to withdraw their participation for any reason at any time during the questionnaire procedure. Arab participants could choose the language of their questionnaire: Arabic or Hebrew. The questionnaires in Arabic were translated into Arabic by a professional translator and then back-translated into Hebrew to assure accuracy.

### 2.3. Measures

Demographic questionnaire: Participants were asked about their gender, age, family status and socio-economic status.

Ways of transmitting messages to minority groups in times of pandemic: This questionnaire was constructed especially for this study and included four sections. Participants were asked to rate each item on a 5-point Likert scale: 1 (*not at all*) to 5 (*very much*). For the purpose of this study, each item was treated separately.

Before the study was conducted, a focus group was gathered that included expert researchers of the study population and members of these communities. The questionnaire was constructed based on the deep discussion that occurred among that group. That is, the questions were outlined ahead of time by the researchers, in light of the need for appropriate policies regarding responses to those questions. The focus group also discussed possible answers to the four questions.

The first section included questions regarding to whom the individual would be most likely to listen with regard to messages about the pandemic. The alternatives were: a family doctor, a doctor who specializes in pandemics, a religious authority, a family authority, a mayor or head of the local authority, the prime minister, the health minister and the director-general of the Health Ministry. The second section related to what information would be important for the individual, in order to follow the instructions. The different items referred to information related to harm to the individual’s health, harm to the health of one’s own children, harm to one’s parents’ health and harm to elders in the extended family, as well as the spread of the virus in one’s own community and the spread of the virus in Israel. The third section related to how participants thought that messages should be delivered. Alternatives were traditional radio and television channels; signs hung on notice boards along the street (*pashkevilim*); Whatsapp, Facebook groups, Twitter, Telegram etc.; messages via HMOs; police loudspeakers in neighborhoods; health ministry loudspeakers and loudspeakers belonging to community organizations. Additionally, the Ultra-Orthodox participants were asked about messages delivered to ‘kosher’ telephones or special Ultra-Orthodox lines (recorded phone messages). The fourth section was focused on when messages should be delivered. Alternatives were in the morning, noon time, evenings, in the beginning of the week, during prayers and over the weekend.

Community sense of coherence [35,36]. This is a 15-item, 7-point Likert-type scale with anchoring phrases at each end. It translates the major themes of Antonovsky’s personal SOC—comprehensibility, manageability and meaningfulness—into community resources. Examples of items are: “To what extent do you feel that you have influence in your community” and “I intend to live in this community in the future.” The mean of the different items was computed to create the scale. Higher scores represent stronger sense of coherence. The Cronbach’s alpha coefficients for the present study were 0.87 for the Ultra-Orthodox group and 0.82 for the Arab group.

### 2.4. Statistical Analysis

First, the prevalence of each response regarding ways of messaging was explored to evaluate the most effective approach in each society. Second, MANOVA was performed to examine the effects of gender, society and interactions of these variables on the various dependent variables. Finally, Pearson correlations were run for each group, separately, followed by a *z*-test to examine significant differences between the correlations observed in the two groups. The software was used for the statistical analyzes SPSS 25.

## 3. Results

Firstly, differences in socio-demographic characteristics were calculated across the two study’s groups:

In this case, 318 individuals from Ultra-Orthodox society and 360 individuals from Arab society filled out a self-reported questionnaire. The age range in each group was 18–67 years (Ultra-Orthodox: *M* = 35.73, *SD* = 12.17; Arab: *M* = 32.77, *SD* = 10.63, *t* = 3.36, *p* < 0.001). Women accounted for 48.4% (*n* = 154) of the Ultra-Orthodox participants and 66.9% (*n* = 241) of the Arab participants (χ^2^ = 23.05, *p* < 0.001). Most of the participants were married: 77.40% of the Ultra-Orthodox participants (*n* = 246) were married and 59.2% of the Arab participants (*n* = 213) were married. In our study, 18.2% of the Ultra-Orthodox participants were single (*n* = 58) and 38.1% of the Arab participants were single (*n* = 137); 4.1% of the Ultra-Orthodox participants were divorced (*n* = 13) and 2.8% of the Arab participants were divorced (*n* = 10). We found that 0.3% of the Ultra-Orthodox participants were widowed (*n* = 1); whereas none of the Arab participants were widowed—χ^2^ = 33.29, *p* < 0.001. In both groups, a large proportion of the participants reported below-average incomes. Specifically, 74.5% of the Ultra-Orthodox participants (*n* = 219) and 59.7% of the Arab participants reported below-average incomes (*n* = 210). Average incomes were reported by 17.3% of the Ultra-Orthodox participants (*n* = 51) and 21% of the Arab participants (*n* = 74). Above-average incomes were reported by 8.2% of the Ultra-Orthodox participants (*n* = 24) and 19.3% of the Arab participants (*n* = 68); χ^2^ = 20.42, *p* < 0.001.

The prevalence of the different responses and community SOC levels within each group are presented in Table 1.

A multivariate analysis of variance was conducted to explore the effects of gender, society (Ultra-Orthodox/Arab) and gender*society interactions on the preferences regarding different ways of messaging. Results are presented for significant effects only. As for gender, significant effects were found for all of the items related to who should deliver the messages: a family doctor (*F*_(1, 666)_ = 11.45, *p* < 0.001, η_p_^2^ = 0.02); a doctor who specializes in pandemics (*F*_(1, 666)_ = 3.70, *p* < 0.01, η_p_^2^ = 0.01); religious authority (*F*_(1, 666)_ = 14.33, *p* < 0.001, η_p_^2^ = 0.02); a family authority (*F*_(1, 666)_ = 9.70, *p* < 0.01, η_p_^2^ = 0.01); a mayor/head of local authority (*F*_(1, 666)_ = 36.62, *p* < 0.001, η_p_^2^ = 0.05); prime minister (*F*_(1, 666)_ = 25.56, *p* < 0.001, η_p_^2^ = 0.04); health minister (*F*_(1, 666)_ = 58.31, *p* < 0.001, η_p_^2^ = 0.08) and the director-general of the Health Ministry (*F*_(1, 666)_ = 31.83, *p* < 0.001, η_p_^2^ = 0.05). Women reported higher ratings for all items and, as noted, the strongest gender effect was related to the delivery of messages by the health minister.

Significant gender effects were also observed for most of the ‘what’ items: individual’s health (*F*_(1, 666)_ = 8.86, *p* < 0.01, η_p_^2^ = 0.01); harm to the health of one’s own children (*F*_(1, 666)_ = 8.01, *p* < 0.01, η_p_^2^ = 0.01); harm to the health of one’s parents (*F*_(1, 666)_ = 12.93, *p* < 0.001, η_p_^2^ = 0.02); harm to the health of elders in the extended family (*F*_(1, 666)_ = 13.83, *p* < 0.001, η_p_^2^ = 0.02) and the spread of the virus in one’s community (*F*_(1, 666)_ = 12.89, *p* < 0.01, η_p_^2^ = 0.02). Similar to the ‘who’ items, women reported higher scores than men for the ‘what’ items. However, all of these gender differences were fairly small.

As for the ‘how’ items, significant gender effects were found for: traditional radio and television channels (*F*_(1, 666)_ = 6.97, *p* < 0.001, η_p_^2^ = 0.004); messages delivered via an HMO (*F*_(1, 666)_ = 15.11, *p* < 0.001, η_p_^2^ = 0.02) and messages delivered via loudspeakers belonging to community organizations (*F*_(1, 666)_ = 8.41, *p* < 0.01, η_p_^2^ = 0.01). The ‘when’ section revealed only one significant gender effect: in the beginning of the week (*F*_(1, 666)_ = 11.64, *p* <.01, η_p_^2^ = 0.02). On all items women scored higher.

Society affected the items related to who should deliver the messages as follows: family doctor (*F*_(1, 666)_ = 36.85, *p* <.001, η_p_^2^ = 0.05); a doctor who specializes in pandemics (*F*_(1, 666)_ = 18.91, *p* < 0.001, η_p_^2^ = 0.03); religious authority (*F*_(1, 666)_ = 432.86, *p* < 0.001, η_p_^2^ = 0.40); a family authority (*F*_(1, 666)_ = 101.67, *p* < 0.001, η_p_^2^ = 0.13); a mayor/ head of local authority (*F*_(1, 666)_ = 65.12, *p* < 0.001, η_p_^2^ = 0.09); prime minister (*F*_(1, 666)_ = 121.17, *p* < 0.001, η_p_^2^ = 0.16); health minister (*F*_(1, 666)_ = 145.32, *p* < 0.001, η_p_^2^ = 0.18) and director-general of the Health Ministry (*F*_(1, 666)_ = 56.47, *p* < 0.001, η_p_^2^ = 0.08). The Ultra-Orthodox participants reported higher scores for all of these items. The society effects were stronger than the gender effects and the most important effects related to religious authority, followed by the health minister, prime minister and family authority.

Significant society effects were also noted for most of the ‘what’ items: individual’s health (*F*_(1, 666)_ = 28.56, *p* < 0.001, η_p_^2^ = 0.04); harm to the health of one’s own children (*F*_(1, 666)_ = 46.67, *p* < 0.001, η_p_^2^ = 0.06); harm to the health of one’s parents (*F*_(1, 666)_ = 22.47, *p* < 0.001, η_p_^2^ = 0.03); harm to the health of elders in the extended family (*F*_(1, 666)_ = 27.39, *p* < 0.001, η_p_^2^ = 0.04); spread of the virus in one’s community (*F*_(1, 666)_ = 27.39, *p* < 0.01, η_p_^2^ = 0.04) and spread of the virus around the country (*F*_(1, 666)_ = 82.15, *p* < 0.001, η_p_^2^ = 0.11). For the ‘what’ items, the Ultra-Orthodox individuals reported higher ratings than the Arab individuals. While small effects were observed for most of the items, the largest effect was noted for the spread of the virus around the country.

As for how the messages should be delivered, significant society effects were found in different directions. For the first two items: traditional radio and television channels (*F*_(1, 666)_ = 29.43, *p* < 0.001, η_p_^2^ = 0.004) and HMO (*F*_(1, 666)_ = 8.67, *p* < 0.01, η_p_^2^ = 0.01), Arab individuals reported higher ratings. However, the Ultra-Orthodox participants rated the various loudspeakers more highly than the Arab participants did: police loudspeakers (*F*_(1, 666)_ = 6.24, *p* < 0.05, η_p_^2^ = 0.01); Health Ministry loudspeakers (*F*_(1, 666)_ = 5.88, *p* < 0.05, η_p_^2^ = 0.01) and loudspeakers belonging to community organizations (*F*_(1, 666)_ = 12.44, *p* < 0.01, η_p_^2^ = 0.02). All of the society effects in this section were very small.

We observed some significant society effects among the ‘when’ items as well. Ultra-Orthodox participants rated most of the options more highly: noon time (*F*_(1, 666)_ = 61.2001, *p* < 0.05, η_p_^2^ = 0.08); evenings (*F*_(1, 666)_ = 9.27, *p* < 0.01, η_p_^2^ = 0.01); in the beginning of the week (*F*_(1, 666)_ = 17.41, *p* < 0.001, η_p_^2^ = 0.03) and during the weekend or on Saturday nights (*F*_(1, 666)_ = 17.47, *p* < 0.001, η_p_^2^ = 0.07). However, ‘during prayers’ was rated more highly by the Arab participants (*F*_(1, 666)_ = 51.57, *p* < 0.001, η_p_^2^ = 0.07). The largest society effects were observed for noon time and during prayers.

Interaction effects of gender*society were less prevalent and appeared only for three items: a mayor/ head of local authority (*F*_(1, 666)_ = 4.23, *p* < 0.05, η_p_^2^ = 0.01); noon time (*F*_(1, 666)_ = 4.48, *p* < 0.05, η_p_^2^ = 0.01) and evenings (*F*_(1, 666)_ = 6.69, *p* < 0.05, η_p_^2^ = 0.01). On all items Ultra-orthodox scored the highest.

Our third question related to the relationships between the preferred ways of messaging and community SOC in each of our groups. Results are presented in Table 2.

Correlations between preferences regarding ‘who’ and ‘what’ items and community SOC appear to be significant and positive in both groups. However, in most cases, they were significantly stronger among the Ultra-Orthodox. A similar trend was observed for the delivery of messages via Health Ministry loudspeakers in neighborhoods. In contrast, three items were positively correlated with community SOC among the Arab population and those relationships were stronger and in the opposite direction to those observed among the Ultra-Orthodox population, among which they were weak and negative: news delivered via traditional media (radio, TV etc.); messages via WhatsApp groups, Facebook groups, Telegram and Twitter; and messages delivered during prayers.

## 4. Discussion

Our study examined the effective ways to disseminate messages about the virus to the Ultra-Orthodox and the Arab communities in Israel during the COVID-19 crisis. Each of these communities has had to cope with the spread of the epidemic in a manner congruent with its own social barriers and behavioral norms.

Our first question asked to whom individuals would be most likely to listen regarding the pandemic. Based on the research literature, we expected that religious leaders would carry great weight in the decision-making of community members in times of crisis and in general. That pattern was observed among the Ultra-Orthodox group, but not among the Arab group. This finding corresponds to the Ultra-Orthodox society and is congruent with the findings of previous studies that have examined the role of religious authorities in the dissemination of health messages to religious groups [22,24]. Within the Ultra-Orthodox community, medical experts also carry significant authority with regard to the dissemination of health messages. It could be that the trends of change within Ultra-Orthodox society have affected this community’s relationships with the medical field and figures from outside the community.

In terms of the role of authority figures in disseminating messages, we found that members of the Arab community first listen to doctors who are experts in infectious diseases and family doctors. The high level of trust that both groups have in the health system is very important for formulating an epidemic-control policy. It should be noted that these two minority groups are both characterized by a low level of trust in state institutions, even though Ultra-Orthodox society seems to be politically represented and Ultra-Orthodox political parties are usually linked to the governing coalition. Attitudes toward the medical system appear to be distinct from attitudes toward the overall political system. Within Arab society, there has been a significant increase in the number of people acquiring higher education [37], especially in the medical professions [38], which may have had an effect on the community’s level of trust in the system.

The second question we sought to answer concerned the types of messages that would most encourage people to adhere to the guidelines. The most effective content reported by the participants in our study was information about the danger of harm to elderly parents and elderly members of the extended family; information regarding risk to the individual was less effective. This finding is congruent with the collectivistic nature of both of these communities and the importance they place on preserving and protecting important others and their community. This was particular important in light of the incidence of COVID-19 among the elderly. The highest death toll has been among people of 80 years and older [39].

We also examined the tools used to disseminate messages among each of the two groups. Our hypothesis that traditional groups would better internalize messages delivered via unique tools that suit their own traditional norms was strongly confirmed among the Ultra-Orthodox community (i.e., loudspeakers in neighborhoods). This finding corresponds with the Ultra-Orthodox way of life, which is characterized by great conservatism. The use of conservative tool will be accepted in all Ultra-Orthodox communities and will not pose a threat to the religious conservatism that is deeply opposed to modernity.

In contrast to the Ultra-Orthodox community, among the Arab community, modern communication tools, television and radio were the best ways to disseminate messages. In Arab society, differing from Ultra-Orthodox society, there has been an accelerated process of modernization. Even though Arab society is still characterized as a society with traditional and religious values, it is possible to reach the Arab community through radio and television, as those forms of media are accepted among that community.

The fourth question referred to the most effective times for disseminating messages. Among the two groups, the most convenient times are evenings and Saturdays. Women were found to be more flexible than men, in terms of the timing of messages.

We also wanted to identify the effects of gender and society on the efficacy of different ways of messaging during the pandemic. The findings show that the women from both groups internalized the messages better and showed more willingness and openness than men to receiving information and cooperating with those authorities that were dealing with the virus in their community. These findings are consistent with the research literature indicating a disparity between women and men in terms of their behavior during a medical crisis [26,27]. Women in these communities are more closely connected to young people and the elderly and it could be that the sense of responsibility that they carry led them to act conscientiously in this time of crisis. Within the Ultra-Orthodox community, the dissemination of messages via women is critically important, in light of the fact that their employment rate is higher than that of their male peers and, therefore, they are more connected to media and have greater exposure to instructions issued during an epidemic.

The same is true of Arab women. They act as primary caregivers and are responsible for the physical and mental health of young people and adults in the family. This puts them in an important position with a particular ability to convey health messages within their community [14].

Finally, we wanted to explore the relationships between the different ways of messaging and community SOC within each of the groups and determine whether those relationships are similar across the two groups. We found that the greater the community SOC, the greater the attention paid to authority figures in each of the two communities. It appears that community SOC is a source of strength for authority figures, particularly among traditional communities, and may help policymakers to transmit messages to minority groups during medical crises and strengthen societal resilience [34]. These findings underscore the importance of maintaining community SOC, which means increasing available resources that foster the feeling for the individual that the community is a safe place for him or her, enabling the individual to predict what will happen when he or she acts in a certain way, as well as the significance of being a part of or affiliated with the specific community. These aspects seem to lead to individuals being more open and receptive to instructions during pandemic.

Community SOC refers to the attitudes and perceptions of community members in relation to the community in which they live, with reference to the way it captures the three components inherent to SOC, namely, comprehensibility, manageability and meaningfulness. The findings show that the higher the level of community SOC, the more weight individuals give to the rulings of their authoritative figures. This factor is extremely important because it impacts policymaking not only in times of crisis, but during any period of change. In general, there is a high level of uniformity that is associated with conservatism and traditionalism [40] and as these communities undergo processes of change and move away from their traditional norms, including social cohesion, traditional figures of authority lose some of their standing in the eyes of community members. There are two sides to this process. On the one hand, it enables the establishment of the individual with the right to challenge authority and express his or her position as he or she wishes. On the other hand, it may create difficulty in changing collective policy as has been the case in the past [7].

### Limitations

Two methodological limitations to this study should be addressed in future research on this subject. First, we propose to conduct a study that will include the entire period of dealing with the virus. The present study referred to the initial period of the outbreak of the virus, but since the pandemic is far from over, monitoring the situation over time should allow us to notice changes in perceptions.

The second recommendation refers to the need for a more fine-grained segmentation of the different streams within the two groups. Within Ultra-Orthodox society, there are several streams that share a common value base but have divergent practices. Arab society is also comprised of different groups and it may be more effective to relate specifically to each group and stream. Given the differences between and within the two populations, it is important examine larger samples, so that it will be possible to look at subgroups within each population.

It is important to note that qualitative research is needed to elucidate different trends in the answers given. For example, Arab participants noted that the Vardi television channels are an effective tool for transmitting messages. It is important to clarify the television channels and radio stations that the Arab population tends to watch and listen to [e.g., Israeli broadcasts, Arab Israeli broadcasts, broadcasts from the Arab world (Al-Jazeera or global).

The COMSOC tools refer to the broader community and lack reference to the family, therefore, future research should include also family members as potential people that can influence individuals with regards to their health care.

## 5. Conclusions

The main conclusion that emerges from this study is that when dealing with minority groups, policymakers should be aware of the processes of change that those groups experience. The two minority groups at the center of this study are currently undergoing significant processes of change and adopting Western norms. This trend has significant implications for the role of professionals, in general, and during a medical crisis, in particular. We see that as traditionalists adopt modern values and integrate both occupationally and socially into general society, they show more trust in professional authority figures. This trust is critical in times of crisis and should be an important consideration in policymaking and the treatment of minority groups during epidemics.

The findings of this study are congruent with the cultural-competency approach, which maintains that during a health crisis, messages to citizens from different minority cultures, especially different religious cultures, should be conveyed in a manner different from that used to communicate with the general public [21]. In religious societies, religious leaders carry significant weight. Therefore, they should be included in the process of formulating instructions for dealing with a crisis, particularly a health crisis. As we found in this work, there is also trust in medical experts and, therefore, such experts should work together with religious leaders.

This finding corresponds with the secular and modern processes that Arab society has undergone, especially in the last decade. These changes permeate and influence fundamental values such as the place of leadership and authority [10]. In the case of the Ultra-Orthodox community in Israel, the partnership of rabbinical leadership with medical leadership would strengthen the public’s faith in the medical system with the backing of rabbinic leadership.

Policymakers should identify the key authority figures in traditional communities and share with them decisions and actions pertaining to the care of the community and its members. Such cooperation establishes trust, reduces community members’ feelings of alienation and helps to establish policies that will be accepted in various communities.

In terms of ways to disseminate messages, the means of dissemination should be specifically tailored to each population and the communication tools most widely used among that population. For Ultra-Orthodox society, the use of traditional tools (i.e., loudspeakers in neighborhoods) has been found to be extremely effective. Among Arab society, one can actually see how applicable the standard tools are. That is, unique fine-tuning is required for each population and messaging strategies should be carefully considered; messaging should not just be carried out according to fixed perceptions that associate traditional groups with the use of traditional tools. Messages should be marketed in ways that correspond to the cultural norms within each group and efforts should be made to identify the people within each community who are best positioned to disseminate those messages. In the context of our work, we found that Ultra-Orthodox and Arab women can serve as agents for disseminating instructions within the family and the community. This is a particularly important resource as it comes from within the community and will not be perceived as the imposition of instructions from the outside.

## Figures and Tables

**Table 1:** Preferences regarding messaging among Ultra-Orthodox and Arab participants.

	Ultra-Orthodox *n* = 318		Arabs *n* = 360	
	*M*	*SD*	*M*	*SD*
Who				
Family doctor	4.38	1.10	3.84	1.30
Specialist (doctor)	4.52	0.95	4.16	1.15
Religious authority	4.61	0.88	2.75	1.36
Family authority	3.97	1.17	3.02	1.27
Mayor	3.75	1.33	3.00	1.34
Prime minister	4.31	1.30	3.38	1.30
Health minister	4.28	1.04	3.26	1.47
Director-general of the Health Ministry	4.36	0.96	3.79	1.26
What				
Risk to my own health	4.53	0.83	4.17	0.97
Risk to my children’s health	4.70	0.76	4.21	1.11
Risk to my parents	4.78	0.63	4.53	0.81
Risk to other elders in the extended family	4.71	0.62	4.42	0.93
Risk of the virus spreading in my community	4.67	0.67	4.37	0.96
Risk of the virus spreading around the country	4.58	0.81	3.79	1.29
How				
News via traditional media—radio, TV, etc.	3.52	1.40	4.06	1.02
Messages via WhatsApp groups, Facebook groups, Telegram, Twitter	2.87	1.48	3.09	1.32
Messages from HMOs	3.50	1.29	3.85	1.13
Police loudspeakers in neighborhoods	3.60	1.27	3.35	1.31
Health Ministry loudspeakers in neighborhoods	3.67	1.26	3.44	1.30
Community organizations’ loudspeakers in neighborhoods	3.65	1.33	3.36	1.45
Messages via ‘kosher’ phones (only for the Ultra-Orthodox)	3.24	1.45	-	-
When				
In the morning	3.15	1.45	3.29	1.40
At noon time	3.79	1.12	3.05	1.22
In the evening	4.52	0.81	4.29	0.94
During prayers	2.53	1.52	3.40	1.40
At the beginning of the week	1.13	1.10	3.80	1.94
Saturday night/During the weekend	4.20	1.10	3.80	1.28
Community sense of coherence	5.04	0.93	4.25	0.91

**Table 2:** Pearson correlations between preferred messaging techniques and community sense of coherence (COMSOC) among Ultra-Orthodox and Arab participants.

	Ultra-Orthodox *n* = 304	Arabs *n* = 360	
**Who**	**COMSOC**	**COMSOC**	***z*-score**
Family doctor	0.15 **	0.22 ***	−0.93
Specialist (doctor)	0.20 **	0.17 **	0.40
Religious authority	0.44 ***	0.32 ***	1.80 *
Family authority	0.28 ***	0.28 ***	0
Mayor	0.34 ***	0.31 ***	0.43
Prime minister	0.28***	0.21 ***	0.95
Health minister	0.28 ***	0.10	2.39 **
Director-general of the Health Ministry	0.34 ***	0.20 ***	1.93 *
**What**			
Risk to my own health	0.29 ***	0.15 **	1.88 *
Risk to my children’s health	0.28 ***	0.17 **	1.43 ^
Risk to my parents	0.22 ***	0.12 *	1.32
Risk to other elders in the extended family	0.31 ***	0.18 **	1.77 *
Risk of the virus spreading in my community	0.32 ***	0.20 ***	1.64 ^
Risk of the virus spreading around the country	0.25 ***	0.18 **	0.94
**How**			
News via traditional media—radio, TV, etc.	−0.10	0.15 **	−3.21 **
Messages via WhatsApp groups, Facebook groups, Telegram, Twitter	−0.01	0.15 **	−2.06 *
Messages from HMOs	0.20 ***	0.14 **	0.80
Police loudspeakers in neighborhoods	0.16 **	0.13 *	0.39
Health Ministry loudspeakers in neighborhoods	0.23 ***	0.10	1.71 *
Community organizations’ loudspeakers in the neighborhoods	0.19 **	0.17 **	0.26
**When**			
In the morning	0.05	0.08	−0.38
At noon time	0.17 **	0.10	0.91
In the evening	0.07	0.06	−0.13
During prayers	−0.01	0.15 **	−2.06 *
At the beginning of the week	0.13 *	0.06	0.90
Saturday night/During the weekend	0.05	0.05	0

^ *p* < 0.06; * *p* < 0.05; ** *p* < 0.01; *** *p* < 0.001.

## Data Availability

Data is available upon request from the corresponding author. The questionnaire will be distributed in response to requests made to the research team.
